# Clinical Efficacy and Safety of Non-Cross-Linked Hyaluronic Acid Combined with L-carnosine for Horizontal Neck Wrinkles Treatment

**DOI:** 10.1007/s00266-021-02307-2

**Published:** 2021-08-10

**Authors:** Shiwei Wang, Huanyun Niu, Yao Liu, Yawen Tan, He Gao, Shuang Ren, Lin Wang

**Affiliations:** 1 Department of Medical Affairs, Imeik Technology Development Co., Ltd, Beijing, China; 2 Department of Medical Cosmetology, Dalian Municipal Central Hospital, No. 42 Xuegong Street, Shahekou District, Dalian, 116003 Liaoning China

**Keywords:** L-carnosine, Horizontal neck wrinkles, Non-cross-linked hyaluronic acid, Wrinkle assessment scale score

## Abstract

*Background* Horizontal neck wrinkle formation is gaining more attention among cosmetic practitioners and clients. To date, hyaluronic acid products are one of the most common treatment options for this aesthetic concern. However, different therapeutic strategies should be given to solve the problem due to multiple etiological reasons. Given that oxidative damage plays a critical role in neck wrinkle formation, anti-oxidative compounds are now considered by physicians when making a treatment plan. *Aims* To evaluate the efficacy and safety of a non-cross-linked hyaluronic acid filler in combination with L-carnosine in treating horizontal neck wrinkles. *Methods* Thirteen patients with a Wrinkle Assessment Scale (WAS) of 2–5 for horizontal neck wrinkles were treated with L-carnosine-containing non-cross-linked hyaluronic acid. Participants were followed-up for 3 months after treatment. The post-treated WAS scores evaluated by physicians were collected when patient satisfaction was surveyed. Any post-treatment adverse events were recorded. *Results* With a single injection of the above filler, the physician-evaluated WAS scores improved by at least one score at one month and the improvement kept consistent as far as three months after injection. According to the last follow-up visit, 11/13 patients were satisfied with the treatment effect of their neck wrinkle. Moreover, adverse events were rare after filler injection, except for local complications that were considered common reactions to the filler injection procedure. *Conclusion *The non-cross-linked hyaluronic acid filler containing L-carnosine is safe and effective for treating horizontal neck wrinkles.

*Level of Evidence IV* This journal requires that authors assign a level of evidence to each article. For a full description of these Evidence-Based Medicine ratings, please refer to the Table of Contents or the online Instructions to Authors www.springer.com/00266.

## Introduction

Multiple factors such as genetic inheritance, vertical head movements, sun exposure and other pathological reasons like neck histological abnormalities are known to cause the appearance of neck wrinkles. Different from facial skin, the local neck derma is more susceptible to environmental factors, owing to less thickness and the small number of sebaceous glands and sweat glands.

Photoaging is one major factor among the external harmful factors. The over-exposure to ultraviolet rays can generate a series of reactive oxygen species (ROS) such as hydroxide ions (OH^−^), superoxide anions (O^2−^), singlet oxygen (^1^O_2_) and hydrogen peroxide (H_2_O_2_) in the local dermal layer. The new generated ROS molecules can induce collagen decrease, which makes the dermal structure collapse to an extent. Meanwhile, the secondary degeneration of elastic fiber can also reduce the elasticity of the dermis, leading to eventual wrinkle formation [1, 2].

Non-surgical techniques such as autologous fat grafting, botulinum toxin injection and hyaluronic acid (HA) filling aimed at correcting the neck wrinkle and rejuvenating neck derma are gaining popularity in current clinical practices. Most physicians recognize the instant effect of HA filling to smoothen the neck horizontal lines, rather than autologous fat grafting, whose main limitation is the low survival rate of lipocytes. Widely applied cosmetic HA fillers are mainly cross-linked, which is less appropriate for neck wrinkles because of its thin dermal layer and less subcutaneous fat tissue. Nodules are reported more often by many physicians after cross-linked HA injection for improving neck wrinkles [3–5].

To maximize efficacy and minimize side effects, non-cross-linked HA seems a better choice for neck wrinkle treatment. Given that the effect duration is a matter of concern with non-cross-linked HA application, different methods of combining other compounds with non-cross-linked HA to extend the local histological repair effect of the wrinkle site have caught scientists’ attention when developing new cosmetic filler products.

L-carnosine, a natural water-soluble dipeptide composed of β-alanine and L-histidine, has been reported to delay cell aging, maintain cell homeostasis, promote wound healing and resist oxidation [6, 7], A non-cross-linked HA product (named as Hearty^®^ in market), mainly containing L-carnosine (2.00 mg/mL), has been developed by Imeik Technology Development Co., Ltd. and been approved by Chinese NMPA (National Medical Products Administration) for correcting mild to severe horizontal neck wrinkle. Besides, low doses of three amino acids (Glycine 0.10 mg/mL, Alanine 0.10 mg/mL, Proline 0.20 mg/mL) and one vitamin (Vit B_2_ 0.005 mg/mL) are also as complementary ingredients in this product. Herein, we investigated the clinical efficacy and safety of this composite HA filler to treat neck horizontal wrinkles.

### Subjects and Methods

#### Patients

A total of 13 patients (12 female and 1 male, mean age: 41 years, range: 26–60 years) with a score of ≥ 2 for horizontal neck wrinkles according to the Wrinkle Assessment Scale (WAS) were recruited in this study (Table 1) [8]. Patients who received other rejuvenation procedures such as laser skin resurfacing, chemical peel or Botox injections within a year were excluded. Other exclusion criteria were being allergic to HA or L-carnosine or any other composite ingredients; taking anticoagulant drugs; local scar formation; infection; and other systematic diseases including severe hypertension, diabetes, obesity (BMI≥30 kg/m^2^), mental health conditions and pregnancy or lactation. All patients signed an informed consent form, and the study was conducted in accordance with the principles of the Helsinki Declaration.

**Table 1:** Description for different grades of horizontal neck wrinkles according to the Wrinkle Assessment Scale (WAS)

Grade	Descriptions
0	No wrinkles
1	Just perceptible wrinkle
2	Shallow wrinkle
3	Moderately deep wrinkle
4	Deep wrinkle, well-defined edges
5	Very deep wrinkle, redundant fold

#### Treatment Procedure

**Fig. 1:**
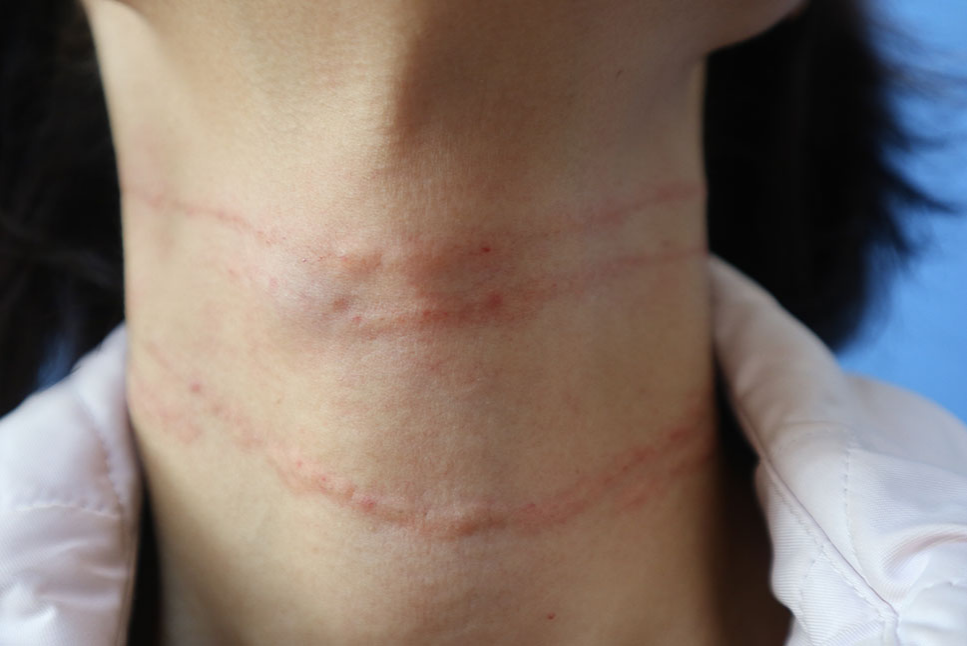
Photograph taken immediately after filler injection treatment. **a** slight strip-shaped bulge was observed

Prior to treatment, patients received local anesthesia with 5 g compound lidocaine cream (25 mg prilocaine + 5 mg lidocaine per gram; Beijing Tsinghua Unisplendour Pharmaceutical Factory, China) on the cervical derma. Then, the neck area of the patients was thoroughly cleaned to rinse off the lidocaine cream. The treatment area—up to mandibular margin and down to the supraclavicular area—was disinfected with iodophor. The patient was then placed in a supine position with the head in an orthostatic position, to ensure normal appearance of the neck wrinkle. The product was injected in a retrograde linear fashion along the neck folds via a 4 mm, 30-gauge needle that was inserted at an angle of 10°–15° and progressed parallel to the skin surface until the entire needle entered into the dermis. After the blood aspiration confirmation to make sure there was no return of blood, the filler was slowly injected while withdrawing the needle. When the injection was completed, the neck folds disappeared and a slight strip-shaped bulge in the dermis was then observed (Figure 1). The average volume of one single injection was about 0.05–0.1 mL. Following all injection procedures, a gentle ice compress was applied to the treated area for 15–30 min to prevent edema and bruising.

#### Efficacy Assessment

Efficacy assessments were performed using WAS, based on standard neck photographs of each patient. Pre-injection, 1-month and 3-month follow-up photographs were, respectively, taken using an SLR camera (Canon EOS M6, Japan). Patients were asked to maintain a fixed head position, and the photographing angle as well as camera parameters such as neck-camera distance, lighting, aperture and flash were kept consistent.

Two physicians independently evaluated the WAS score for each pre-, 1-month and 3-month post-treatment photograph, which was randomized after the 3-month follow-up. In case of a discrepancy on the WAS score, a third physician re-evaluated the score and settled the disagreement. The post-treatment improvement of WAS score was hence calculated to reveal the efficacy of the filler treatment.

Moreover, patients’ satisfaction was surveyed at each follow-up visit using a 5-grade classification from 0 (very dissatisfied) to 4 (very satisfied). A grade of ≥3 was considered satisfactory, and the satisfaction rate was calculated as the total number of satisfied cases divided by the total case number.

#### Safety Assessment

Any procedure-related adverse event (AE) such as injection-site pain, redness, bruising or edema was recorded.

#### Statistical Analysis

A Wilcoxon signed-rank test was performed to compare the pre- and post-treatment WAS scores. *P*<0.05 was considered to indicate statistical significance.

## Results

In the pre-treatment WAS evaluation of the 13 patients, two patients scored 5, while six, four and one patients scored 4, 3 and 2, respectively, which were recorded as the baseline scores. With a single treatment, the injection volume for each patient to smoothen their neck horizontal wrinkles ranged from 1.2 mL to 1.5 mL. Demographics, interventions and effective assessments data are presented in Table 2.

**Table 2:** Overview of demographics, interventions and effective assessments data

Patient No.	Age (years)	Sex	Dose (mL)	WAS Score			Patient Satisfaction*
				Pre-treatment	1-month post-treatment	3-month post-treatment	
1	38	F	1.3	3	2	2	3
2	36	F	1.5	4	3	3	3
3	55	F	1.5	4	4	N/A	2
4	27	F	1.5	4	3	N/A	3
5	60	M	1.5	5	3	N/A	4
6	32	F	1.5	3	2	2	3
7	55	F	1.5	5	4	N/A	2
8	32	F	1.4	3	2	2	4
9	54	F	1.5	4	3	N/A	4
10	26	F	1.2	2	1	1	4
11	42	F	1.5	4	3	3	4
12	43	F	1.3	3	2	2	4
13	33	F	1.5	4	2	2	4

*F: Female; M: Male; N/A: not available.*

^*^: Scores of the last visit were presented in the column of “Patient Satisfaction.”

**Fig. 2:**
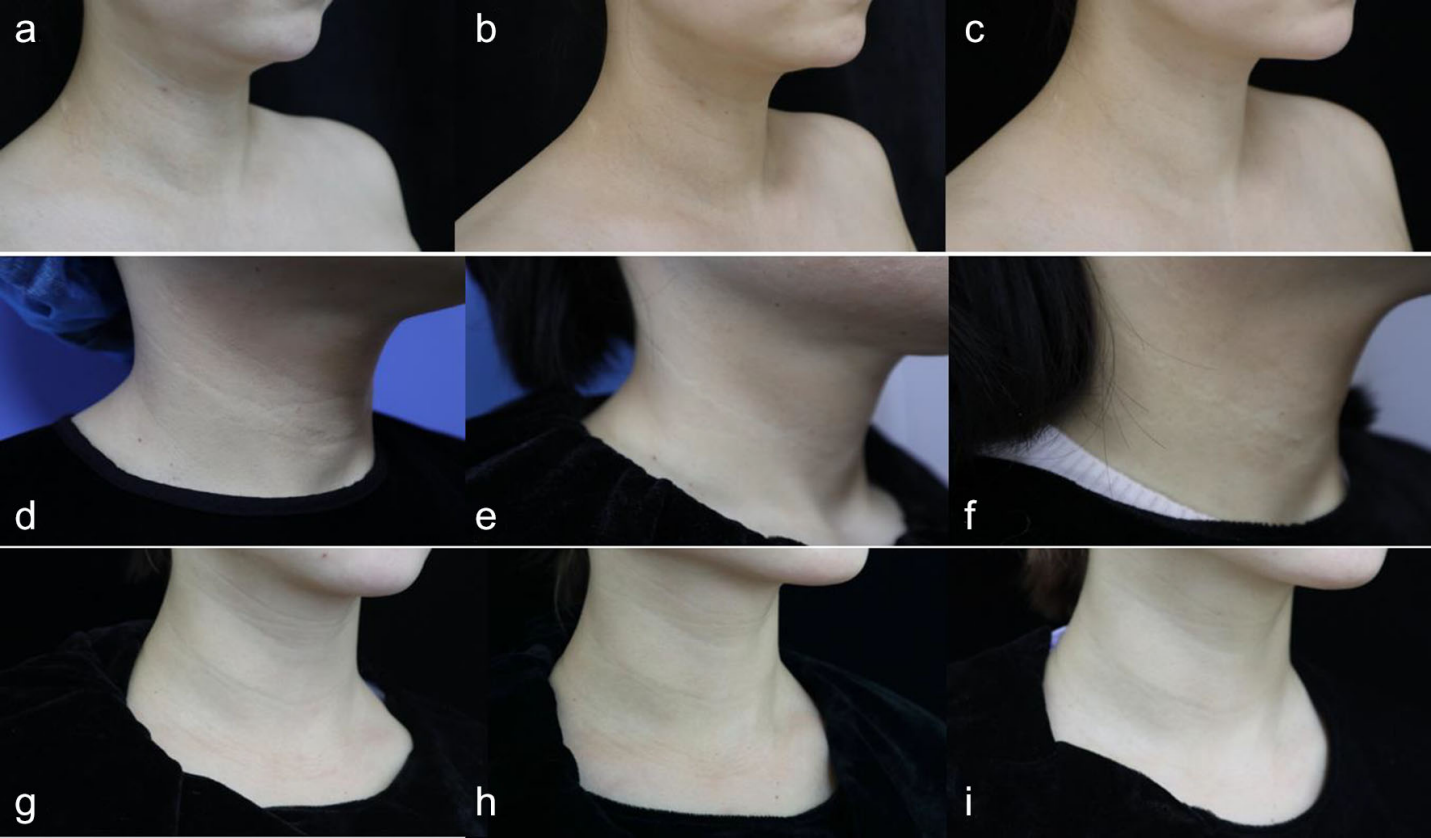
Clinical pictures of patient 8 (**a**-**c)**, patient 10 (**d**-**f**), patient 12 (**g**-**i**). **a**, **d**, **g**: pre-treatment; **b**, **e**, **h**: 1-month post-treatment; **c**, **f**, **i**: 3-month post-treatment

**Fig. 3:**
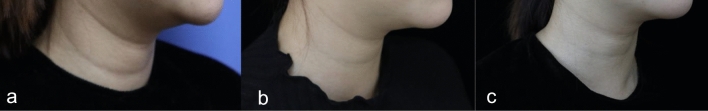
Clinical pictures of patient 13. **a**: pre-treatment; **b**: 1-month post-treatment; **c**: 3-month post-treatment

The one-month follow-up was completed by all patients. According to physicians’ evaluation, 12/13 patients received at least one-level improvement. (Figure 2) The median (Q1, Q3) WAS score decreased from 4 (3, 4) at baseline to 3 (2, 3) post-treatment, which was statistically significant (*P*<0.001). In addition to this, 2/12 patients achieved a 2-level improvement of WAS score with obvious refining of skin texture after a single injection. (Figure 3)

However, one patient did not achieve any WAS score improvement at the 1-month follow-up. This result was likely attributed to the patient’s older age and rather low skin texture due to severe photoaging damage than other younger patients.

In eight patients who completed the 3-month follow-up visit, all WAS scores were consistent with those at the 1-month follow-up, indicating the filling efficacy could last as far as 3 months after a one single injection.

With respect to the patient satisfaction survey, 11 patients were satisfied with the treatment effect at the last follow-up visit, achieving a satisfactory rate as 84.61%; of these, four reported grade 3 and seven were grade 4 (very satisfied). However, the remaining two patients were grade 2, showing dissatisfaction. This is likely because of their severe neck wrinkles (scored as 5 and 4, respectively) and the weakness of dermal elasticity of the neck skin.

Common and mild AEs like injection site pain (*n*=1), redness (*n*=3), bruising (*n*=2) and swelling (*n*=1) were observed during and/or right after injection. Most of them disappeared within one week of the procedure without any specific treatment other than for gentle ice compression, per physician’s advice. No patients reported allergic symptoms in either the injection site or other distant locations. During the 3-month follow-up period, no other systematic AEs or severe AEs were reported.

## Discussion

In this study, we found satisfactory effects for the treatment of neck horizontal wrinkles by non-cross-linked HA mainly combined with L-carnosine. Statistical analysis showed a significant improvement of WAS score at 1-month post-treatment compared to that at baseline (*P*<0.001). In addition, the WAS scores of eight patients were consistent during the 3-month follow-up period, showing that the clinical efficacy of this filler could last over 3 months. Evaluation of patient satisfaction revealed a high satisfactory rate (11/13, 84.61%) based on the last follow-up data of all subjects.

Less occurrence of AEs could be a reason of patients’ high satisfaction. Patients only reported mild bruising, pain and redness following the filler injection, which are widely considered the common AEs of filler injection. Moreover, all treatment-related local AEs lasted only for a short duration (7 days) and were resolved with a gentle ice compress, not needing any further medical intervention.

The efficacy results within 3 months of one single treatment are more impressive than our general understanding of non-cross-linked HA injection, whereas being consistent with other clinical reports of using cross-linked HA. Tseng *et al.* showed that the score based on a horizontal neck fold severity scale improved after 1 month of the first injection, while the improvement peaked at 2 and 4 weeks after a single touch-up following the first dose, one month later. After that period, the improvement slightly diminished but was still significant from baseline till the end of follow-up [9].

In contrast, the AEs observed in our study were much less severe than those usually seen with cross-linked HA application. The cross-linking process is to increase elastic modulus G' or cohesivity and slow down degeneration to realize a long-term effect. An accompanying side effect of cross-linked HA filler is nodule formation, especially in case of injection into the superficial dermal layers [10]. Due to the thin skin and lack of adipose tissue, the nodule risk would be highly increased when patients receive cross-linked HA filling for re-surfacing the horizontal neck wrinkle. Therefore, we prefer to use the non-cross-linked HA-based filler for neck rejuvenation to reduce complications. However, the effectiveness of non-cross-linked HA filling is hardly lasted, which limits its clinical application.

To our knowledge, many studies have demonstrated the relationship between photoaging and formation of horizontal neck lines. Long-wave and mid-wave ultraviolet (UVA and UVB) rays could pass through the epidermis as deep as the dermal layer, causing multiple sunburned reactions like erythema, hyperpigmentation and skin aging. Mechanically, the excessive accumulation of free radicals such as OH^-^ and O^2-^ generated by over-exposure to UVA/UVB may result in a series of non-selective oxidative damages to cell membranes, proteins and nucleic acids [2, 11]. In addition, new-generated advanced glycation end products (AGEs) can result in a decrease in tissue permeability by forming cross-linked products of collagen and elastin and decreasing the local exchange of nutrients and metabolites, which can eventually lead to reduced skin elasticity and wrinkle formation [12].

To target these secondary damages, we noticed this non-cross-linked HA filler combined with a main ingredient of L-carnosine, a natural dipeptide composed of β-alanine and L-histidine. We speculated that it could eliminate the overloaded free radicals and AGEs, as the UVA/UVB exposure would consistently affect the local site after first injection treatment. Many studies reported the effects of L-carnosine on free radicals and as a therapeutic agent in a wide range of pathologies, such as wound healing, hypertension and cerebral vascular diseases [13–16]. Besides, in respect of various pathologies, multiple pathways, e.g., oral, intravenous, intramuscular, subcutaneous and intracranial administration, have been proved effective and safe with L-carnosine treatment [15–19]. Specifically, Narda *et al.* applied *ex-vivo* a novel facial cream containing carnosine in human skin explants and found it can significantly reduce levels of AGEs in both epidermis and reticular layer of dermis [20]. Our results showed that the effect of the filler injection evaluated via WAS score was maintained at the end of the 3-month follow-up, proving that L-carnosine had a positive role in protecting the treated neck wrinkle from further oxidative damage. This could likely provide a much more conducive environment for tissue repair processes such as collagen regeneration and realigning of elastic fibers, even if the non-cross-linked HA maybe degenerated in under a month, as anticipated. Moreover, the low-dose proline, glycine and alanine play roles as amino acid materials for local collagen synthesis, while vit B_2_ could physically modulate cellular activities during repairing process [21].

Considering the efficacy and safety together, we believe this combination product prevents treatment-related complications and actively prolongs the efficacy of the filler by improving local tissue protecting and repairing activity. Theoretically, to realize a stronger validation of L-carnosine’s effectiveness, a randomized controlled trial with a pure non-cross-linked HA as control arm will be highly recommended. However, being restricted by the lack of this kind of product, it was difficult to design this study as a parallel comparative one, to better investigate L-carnosine’s function. Besides, the small patient sample size and short follow-up duration are also limitations of the study. Further studies should be conducted to investigate a long-term effectiveness of single injection as well as whether a much longer effect could be realized by the touch-up treatment. We also acknowledge the limitation of lack of device-based quantification assessment for effectiveness evaluation in this study, such as high-resolution photographing and digital measurement. Nevertheless, the WAS scoring system is a widely used assessment scale for most facial and horizontal neck fold treatment, and we aimed to be as objective as possible by ensuring that physicians independently evaluated the results after photo randomization. Another limitation of the study is the lack of efficacy difference analysis with respect to different levels of WAS score, which may help to further explore the relationship between the severity of neck wrinkles and treatment dose.

According to above hypothesis, this composite filler targets the main reason of neck horizontal wrinkle formation as well as skin aging resulting from excessive ultraviolet exposure. Due to its safety of superficial application, it is surely interesting that whether the product could be used as a skin booster for neck rejuvenation. We expect to conduct a related study in the future.

In conclusion, our results show the short-term efficacy and safety of non-cross-linked HA combined with L-carnosine for mild-to-severe horizontal neck wrinkle. The non-cross-linked HA could immediately smoothen the neck folds and L-carnosine could possibly prolong the duration of efficacy possibly by reducing the UV-related damage and promoting the collagen regeneration, despite the HA degenerating shortly after injection. However, further studies are required to investigate the long-term effectiveness of this combination filler, and more data are needed to determine the optimal treatment strategy for neck horizontal wrinkles as well as neck skin aging.
